# Depressive Symptoms Have Distinct Relationships With Neuroimaging Biomarkers Across the Alzheimer’s Clinical Continuum

**DOI:** 10.3389/fnagi.2022.899158

**Published:** 2022-06-20

**Authors:** Inès Moulinet, Edelweiss Touron, Florence Mézenge, Sophie Dautricourt, Vincent De La Sayette, Denis Vivien, Natalie L. Marchant, Géraldine Poisnel, Gaël Chételat

**Affiliations:** ^1^Physiopathology and Imaging of Neurological Disorders (PhIND), Institut National de la Santé et de la Recherche Médicale, Blood and Brain @ Caen-Normandie, GIP Cyceron, Université de Caen Normandie, Caen, France; ^2^CHU de Caen, Service de Neurologie, Caen, France; ^3^Département de Recherche Clinique, CHU de Caen-Normandie, Caen, France; ^4^Division of Psychiatry, University College London, London, United Kingdom

**Keywords:** Alzheimer’s disease, amyloid deposition, cognition, depressive symptoms, anxiety, glucose metabolism, subjective cognitive decline, gray matter

## Abstract

**Background:**

Depressive and anxiety symptoms are frequent in Alzheimer’s disease and associated with increased risk of developing Alzheimer’s disease in older adults. We sought to examine their relationships to Alzheimer’s disease biomarkers across the preclinical and clinical stages of the disease.

**Method:**

Fifty-six healthy controls, 35 patients with subjective cognitive decline and 56 amyloid-positive cognitively impaired patients on the Alzheimer’s continuum completed depression and anxiety questionnaires, neuropsychological tests and neuroimaging assessments. We performed multiple regressions in each group separately to assess within group associations of depressive and anxiety symptoms with either cognition (global cognition and episodic memory) or neuroimaging data (gray matter volume, glucose metabolism and amyloid load).

**Results:**

Depressive symptoms, but not anxiety, were higher in patients with subjective cognitive decline and cognitively impaired patients on the Alzheimer’s continuum compared to healthy controls. Greater depressive symptoms were associated with higher amyloid load in subjective cognitive decline patients, while they were related to higher cognition and glucose metabolism, and to better awareness of cognitive difficulties, in cognitively impaired patients on the Alzheimer’s continuum. In contrast, anxiety symptoms were not associated with brain integrity in any group.

**Conclusion:**

These data show that more depressive symptoms are associated with greater Alzheimer’s disease biomarkers in subjective cognitive decline patients, while they reflect better cognitive deficit awareness in cognitively impaired patients on the Alzheimer’s continuum. Our findings highlight the relevance of assessing and treating depressive symptoms in the preclinical stages of Alzheimer’s disease.

## Introduction

Neuropsychiatric symptoms (NPS) are present in 90% of patients with Alzheimer’s disease (AD) dementia (Finkel et al., 1996), with depressive and anxiety symptoms being two of the most common NPS (Banning et al., 2019). They are associated with decrease in quality of life, caregiver’s burden, and increased institutionalization (Gilley et al., 2004; Hoe et al., 2006, 2007). In cognitively unimpaired individuals, it has been shown that depressive and anxiety symptoms, even at a subclinical level, increase the risk of cognitive decline (Geda et al., 2014; Zhang et al., 2020) and of developing AD (Harrington et al., 2015; Petkus et al., 2016). With regards to the cognitive and brain substrates of depressive and anxiety symptoms, studies are sparse and findings are mixed. In patients with AD dementia, one previous study found that patients with persistent neuropsychiatric symptoms had worse cognitive outcomes (Poulin et al., 2017). Regarding neuroimaging biomarkers, some studies found that depressive and anxiety symptoms were associated with more AD neuroimaging hallmarks in either Mild Cognitive Impairment (MCI) or AD patients (Chen et al., 2021; Mendez, 2021), such as lower gray matter volume (GM) (Son et al., 2013; Dhikav et al., 2014; Fujishima et al., 2014; Lebedeva et al., 2014; Tagai et al., 2014; Hayata et al., 2015; Wu et al., 2020), lower glucose metabolism and/or perfusion (Hashimoto et al., 2006; Levy-Cooperman et al., 2008), and higher amyloid deposition (Brendel et al., 2015; Bensamoun et al., 2016; Krell-Roesch et al., 2019). However, conflicting results have been reported in other studies showing a link between depressive symptoms and higher gray matter volume (Auning et al., 2015; Enache et al., 2015), glucose metabolism and/or perfusion (Tagai et al., 2014; Auning et al., 2015; Brendel et al., 2015; Tommaso et al., 2016) or no association between psychoaffective factors and neuroimaging markers (Horínek et al., 2006; Starkstein et al., 2009; Serra et al., 2010; Poulin et al., 2011; Mori et al., 2014; Chung et al., 2015; Bensamoun et al., 2016; Huey et al., 2017; Banning et al., 2019). Moreover, studies that assessed these relationships in patients with subjective cognitive decline (SCD), i.e., cognitively unimpaired individuals, who are concerned that they have reduced cognitive function (Jessen et al., 2020), are sparse and reported mixed findings. Thus, higher depressive symptoms were associated with both lower (Enache et al., 2015) or higher (Auning et al., 2015) hippocampal gray matter volume in SCD patients.

Given the impact of depressive and anxiety symptoms on quality of life and even prognosis, improving our knowledge on their cognitive and brain substrates across the clinical continuum from normal cognition to Alzheimer’s dementia is particularly relevant for clinical management and AD risk reduction. To date, the existing literature on this research field showed inconsistencies between studies especially regarding the direction of the neuroimaging findings. Furthermore, to our knowledge, no study included cognitive measures and complementary multimodal neuroimaging data throughout the Alzheimer’s clinical continuum at the same time. Therefore, this study aims at providing a comprehensive assessment of the links between depressive and anxiety symptoms, and cognition as well as multiple measures of brain integrity, throughout the clinical continuum from normal cognition to Alzheimer’s dementia, to further our understanding of the relevance and mechanisms of psychoaffective factors in preclinical and clinical AD. We hypothesized that depressive and anxiety symptoms would be associated with AD-related cognitive and brain alterations in both preclinical and later stages.

## Materials and Methods

### Participants

All participants were recruited as part of the *Imagerie Multimodale de la Maladie d’Alzheimer à un Stade Précoce* (IMAP +) Study in Caen, France. Ninety-one patients and 56 controls above 50 years old were included, all living at home, and with no history or clinical evidence of neurologic or psychiatric disorder, alcohol use disorder or drug abuse. Notably, none of the participants met diagnostic criteria for major depression or anxiety disorder, as defined in the Diagnostic and Statistical Manual of Mental Disorders, Fourth Edition. The inclusion and group classification of the participants were based on a clinical interview and a standardized neuropsychological assessment (including tests of episodic memory, working memory, language skills, executive functions, and visuospatial abilities), according to internationally agreed criteria [see details in (La Joie et al., 2012, 2016; Perrotin et al., 2017)]. Patients were either patients with SCD (*n* = 35) or patients on the Alzheimer’s continuum (ADC patients; *n* = 56) and were all recruited from local memory clinics. SCD patients reported memory complaints and showed normal performance in all tests of the standardized neuropsychological assessment. During the interview, the clinician ensured that the complaint was not related to current medication or medical condition, and did not fulfill NINCDS-ADRDA criteria for probable AD (McKhann et al., 1984).

ADC patients were all selected to be positive for amyloid (see Section “Neuroimaging data and processing” for details). They included patients with MCI selected based on Petersen’s criteria (*n* = 28) (Petersen and Morris, 2005) and patients with dementia fulfilling NINCDS-ADRDA clinical criteria for probable AD (*n* = 28) (McKhann et al., 1984). Clinical diagnosis was assigned by consensus under the supervision of a senior neurologist and neuropsychologists. The SCD group consisted of both amyloid positive and amyloid negative participants (amyloid negative *n* = 23; amyloid positive *n* = 4; florbetapir-Positron Emission Tomography (PET) images not available *n* = 8). Finally, the 56 healthy elderly control subjects were recruited from the community, performed in the normal range on all tests from the standardized neuropsychological assessment, and were all selected to be amyloid negative (see below).

IMAP + was approved by the regional ethics committee (Comité de Protection des Personnes Nord-Ouest III) and is registered with ClinicalTrials.gov (number NCT01638949). All participants gave written informed consent before the examinations.

### Psychoaffective Assessment

The Montgomery-Åsberg Depression Rating Scale (MADRS) (Montgomery and Asberg, 1979), a clinician-administered 10-item questionnaire with scores ranging from 0 to 60, was used to assess depressive symptoms at the time of the evaluation. The Spielberger State-Trait Anxiety Inventory form Y-A (STAI-A) (Spielberger et al., 1970), a 20-item self-rated questionnaire with scores ranging from 20 to 80, assessed state anxiety symptoms at the time of the evaluation. For both scales, higher scores indicated higher levels of symptoms of depression and anxiety. All participants were screened so as not to meet diagnostic criteria for major depression or anxiety disorders (see exclusion criteria above), but our goal was to assess depressive and anxiety symptoms on a continuum below this threshold for disorder (Altman and Royston, 2006; Laborde-Lahoz et al., 2015). Therefore, these scores were used as continuous variables in all analyses (see Section “Statistical analysis”) instead of classifying patients as presenting or not depressive/anxiety symptoms or disorders.

### Cognitive Assessment

Global cognition was measured using the Mini Mental State Examination (Folstein et al., 1975) (MMSE, scores from 0 to 30). Verbal episodic memory was assessed using the Encoding, Storage and Recuperation (ESR) word list free recall sub-score (Eustache et al., 2015), consisting on the recall of two distinct 16-word lists after either a superficial or a deep encoding phase. The final score resulted from the sum of the superficial and deep encoding sub-scores (scores from 0 to 32).

### Neuroimaging Data and Processing

All participants underwent neuroimaging scans on the same Magnetic Resonance Imaging (MRI) and PET scanners at the Cyceron Centre (Caen, France), as previously described in detail (La Joie et al., 2012; Besson et al., 2015), within 3-month interval from the psychoaffective and cognitive assessments. We measured GM volume with MRI, brain glucose metabolism with 18F-fluorodeoxyglucose (FDG)-PET and amyloid deposition with florbetapir-PET. The detailed acquisition procedure is available in the Supplementary Material 1. Global GM volumes from the MRI and neocortical standardized uptake value ratio (SUVr) from the PET scans were extracted as described below and used in the following analyses as continuous variables.

All neuroimaging pre-processing steps were performed with the Statistical Parametric Mapping version 12 (SPM12) software (Wellcome Trust Centre for Neuroimaging, London, United Kingdom). Briefly, T1-weighted MRI images were segmented and spatially normalized to the Montreal Neurological Institute (MNI) space and non-linear warping effects on volumes were corrected by modulating the resulting normalized GM segments (Chételat et al., 2008; Villain et al., 2008; La Joie et al., 2012).

PET images were corrected for partial volume effects using the Müller-Gärtner method (Müller-Gärtner et al., 2016). Resulting images were coregistered onto their corresponding MRI, normalized with the deformation parameters used for the MRI procedure, and then quantitatively normalized using the cerebellar GM as the reference region (Chételat et al., 2008; Villain et al., 2008; La Joie et al., 2012; Bejanin et al., 2019).

For each participant, a total GM volume and global FDG-PET value was calculated by applying a binary mask of GM (including voxels with a GM probability > 30% excluding the cerebellum) on the corresponding preprocessed images. The global neocortical SUVr was obtained from the florbetapir-PET images using a neocortex mask (including all regions but the cerebellum, hippocampus, amygdala and subcortical gray nuclei), as described in detail elsewhere (La Joie et al., 2012). This global neocortical SUVr was used as a continuous variable for further analyses (see below) and also to classify subjects as positive or negative for amyloid. This classification was used to select amyloid-negative HC and amyloid-positive MCI/AD patients. The threshold was calculated from a group of 45 young participants from the IMAP project (between 20 and 40 years old) using the mean + 2SD, corresponding to a SUVr of 1.02 under which participants were considered amyloid negative and above which patients were considered amyloid-positive (Besson et al., 2015; Perrotin et al., 2017).

### Statistical Analysis

#### Main Analysis

To assess the differences in psychoaffective factors across clinical groups, we conducted analyses of covariance (ANCOVA) with education, age and sex as covariates and then performed *post hoc* tests to assess group-differences when a main effect of the clinical group was found. We also performed multiple regressions to assess within group associations between psychoaffective measures (i.e., depressive and anxiety symptoms) on the one hand, and cognition (global cognition and episodic memory) and neuroimaging data (GM volume, glucose metabolism and amyloid load) on the other hand. Results are presented with correction for level of education, age and sex. When a significant association was found with either depressive or anxiety symptoms, then the same analysis was repeated adding the other psychoaffective score (anxiety or depressive symptoms, respectively) as an additional covariate to further test whether the association was specific to the one and independent from the other. Indeed, these two symptoms are known to be related and found to be correlated in the present study in the ADC patient group (Spearman’s correlation *p* = 0.01, *r* = 0.356). Pairwise deletion was used in case of missing data and all statistical analyses were performed using the STATISTICA software (v13.0, StatSoft Inc., Tulsa, OK, United States).

### Supplementary Analyses

Scores of depressive and anxiety symptoms were not normally distributed; thus, correlation analyses were repeated with non-parametric Spearman’s correlation tests. The score for depressive symptoms also showed an important floor effect with a large number of participants having a score of zero. To check that the results were consistent and not driven by the floor effect, we repeated all analyses regarding depressive symptoms excluding these participants, i.e., within subgroups of participants with at least one depressive symptom (*n* = 21 HC, *n* = 23 SCD, *n* = 42 ADC patients).

## Results

### Group Characteristics

Group characteristics are listed in Table 1.

**TABLE 1:** Study sample characteristics.

	HC	n	SCD	n	ADC patients	n	ANCOVA *p*-value	*Post hoc* Tukey-test *p*-value
Sex, *n* (M/F)	56 (25/31)		35 (18/17)		56 (31/25)		NS (χ*^2^*)	
Level of education, years	12.64 (3.82)	56	13.43 (3.11)	35	11.30 (3.71)	56	**0.013**	HC, SCD > ADC (*p* = *0.074*, ***p* = 0.016**)
Age, years	69.75 (5.61)	56	67.51 (6.85)	35	71.18 (8.65)	56	*0.075*	SCD < ADC (*p* = *0.060*)
Psychoaffective measures								

Depressive symptoms	0.85 (1.34)	55	3.80 (4.44)	35	3.62 (3.68)	55	**< 0.001**	HC<SCD, ADC (**both *p* < 0.001**)
Anxiety symptoms	27.85 (8.09)	48	27.17 (8.90)	29	31.08 (11.01)	53	*0.080*	NS, no trend
Cognitive measures								

Global cognition	29.00 (1.08)	54	28.88 (1.05)	33	23.71 (4.80)	56	**< 0.001**	HC, SCD > ADC (**both *p* < 0.001**)
Episodic memory	14.39 (3.37)	56	14.34 (3.73)	35	5.84 (2.52)	50	**< 0.001**	HC, SCD > ADC (**both *p* < 0.001**)
Neuroimaging measures								

GM volume	0.61 (0.06)	53	0.63 (0.05)	28	0.58 (0.06)	56	**< 0.001**	HC, SCD > ADC (***p <* 0.001, *p* = 0.002**)
Glucose metabolism	1.08 (0.07)	49	1.13 (0.07)	27	0.99 (0.08)	55	**< 0.001**	HC, SCD > ADC (**both *p* < 0.001**) HC < SCD (***p* = 0.035**)
Amyloid load	0.88 (0.06)	43	0.96 (0.18)	27	1.49 (0.30)	56	**< 0.001**	HC, SCD < ADC (**both *p* < 0.001**)

*Values indicate mean (standardized deviation) unless otherwise stated. Between-group differences for demographic variables were assessed using ANCOVA for continuous variables (corrected for sex, as well as age or level of education, respectively) and χ^2^ tests for categorical variables. All other ANCOVA were corrected for the level of education, age and sex, comparing HC, SCD and ADC patients. Values in bold correspond to significant p values (p < 0.05) and values in italic correspond to trends (0.05 < p < 0.1). ADC Alzheimer’s continuum ANCOVA, Analysis of covariance; GM, Gray matter; HC, Healthy controls; NS, Not significant; SCD, Subjective cognitive decline.*

### Psychoaffective Factors and Their Links With Cognition and Neuroimaging Biomarkers

**FIGURE 1:**
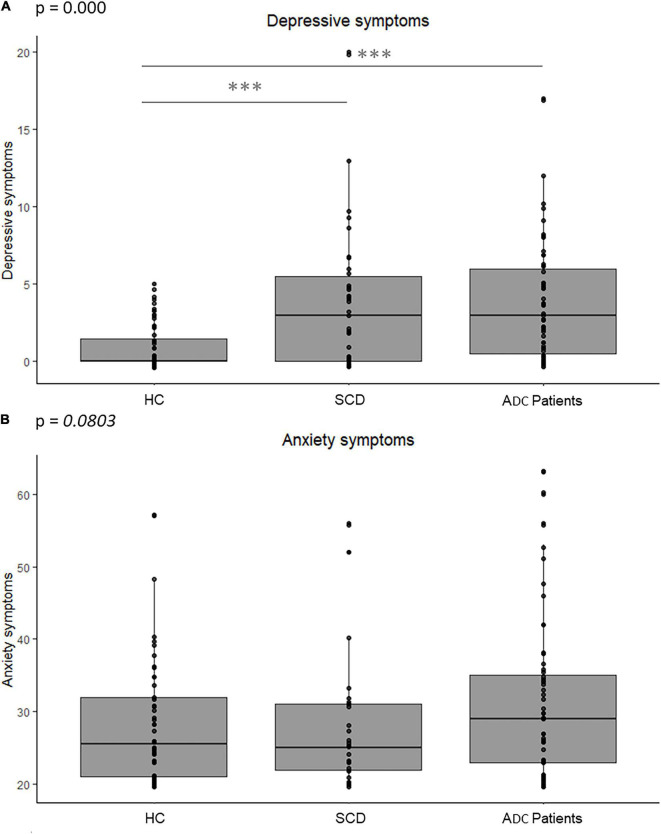
Difference in psychoaffective factors between groups of healthy controls, SCD and ADC patients. Graphs indicate boxplots of depressive and anxiety symptoms in each group. The *p* value of the main effect of group, measured with ANCOVAs corrected for the level of education, age and sex, is indicated on the graph. Significant *post hoc* results between each group are indicated with stars, ***corresponding to *p* values (*p* < 0.001). **(A)** ANCOVA on depressive symptoms. **(B)** ANCOVA on anxiety symptoms. *ADC*, Alzheimer’s continuum; *ANCOVA*, Analysis of covariance; *HC*, Healthy controls; *p, p* value; *SCD*, Subjective cognitive decline.

With regard to psychoaffective factors, depressive symptoms significantly differed across groups (Table 1 and Figure 1A) even after accounting for anxiety symptoms (*p* < 0.001). *Post hoc* analyses showed that patients, either SCD or ADC patients, had more depressive symptoms than HC, but did not differ from each other (HC-SCD *p* < 0.001; HC-ADC patients *p* < 0.001; SCD-ADC patients *p* = 0.9). Regarding anxiety symptoms, we found a trend of group difference (Table 1 and Figure 1B) that did not remain when also correcting for depressive symptoms (*p* = 0.1). However, *post hoc* analyses revealed no significant between-group differences (HC-SCD *p* = 0.922; HC-ADC patients *p* = 0.144; SCD-ADC patients *p* = 0.129).

**FIGURE 2:**
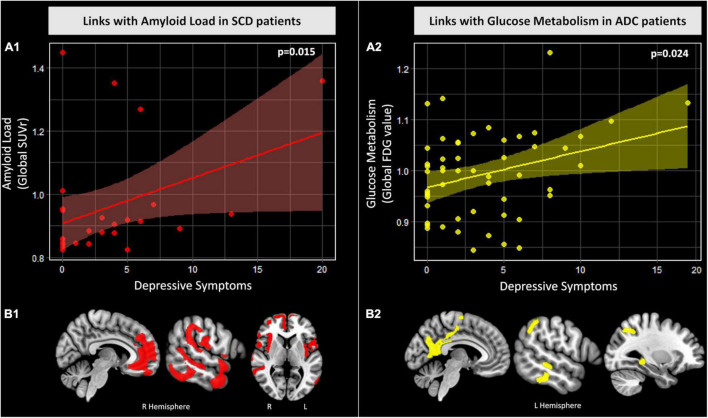
Relationships between depressive symptoms and amyloid load in SCD patients and glucose metabolism in ADC patients. (A) Graphs of the associations between depressive symptoms and global measures of amyloid load in SCD patients (A1) and glucose metabolism in ADC patients (A2). Reported statistics were obtained from the multiple linear regressions corrected for level of education, age and sex. Raw values are plotted and linear trends and confidence intervals (95%) are represented for SCD patients (red) and ADC patients (yellow). (B) Results of the voxelwise multiple regressions between depressive symptoms and amyloid deposition in SCD patients [(B1) red] and glucose metabolism in ADC patients [(B2) yellow]. Analyses were corrected for level of education, age and sex. All results are presented at a p_uncorrected_ < 0.005 threshold combined with a minimum cluster size determined by Monte-Carlo simulations to achieve a corrected statistical significance of *p* < 0.05 (i.e., k_*corrected*_ ≥ 1476 voxels for amyloid deposition and k_*corrected*_ ≥ 1757 voxels for glucose metabolism). Abbreviations *ADC*, Alzheimer’s continuum; *FDG*, 18F-fluorodeoxyglucose; *L*, Left; *p, p* value; *R*, Right; *SCD*, Subjective cognitive decline; *SUVr*, Standardized uptake value ratio.

The results of the analyses assessing the links between depressive or anxiety symptoms on the one hand, and cognitive or neuroimaging measures on the other hand, are reported in Table 2. Higher depressive symptoms were associated with higher amyloid load in the SCD group (Figure 2A1). In contrast, in the group of ADC patients, higher depressive symptoms were associated with better episodic memory performance and higher glucose metabolism (Figure 2A2) and tended to be related to a better global cognition. We found the same results when also correcting for anxiety symptoms, but the association with global cognition was no longer a trend (*p* = 0.1; see Supplementary Table 1). Non-parametric analyses showed a similar pattern of results, though less strong (see Supplementary Table 2). We also found the same results as in our main analyses in the subgroup with at least one depressive symptom, except for the association between depressive symptoms and episodic memory performance in the ADC patient group, which was no longer significant (see Supplementary Table 3). Finally, to test whether the link between depressive symptoms and amyloid load was specific to the SCD stage, or reflected the greater variability of the amyloid measure in the SCD group as it included both amyloid-positive and amyloid-negative patients, we repeated this analysis in a group of MCI + AD that included both amyloid-positive and amyloid-negative patients (see Supplementary Table 4 for further details). We found no correlation (*p* = 0.9, *r* = 0.01) between depressive symptoms and amyloid load in this group.

**TABLE 2:** Relationships of psychoaffective factors to cognition and neuroimaging measures.

Depressive symptoms	HC	SCD	ADC patients
Cognitive measures			

Global cognition	0.358 (−0.139)	0.404 (0.165)	*0.065* (*0.272)*
Episodic memory	0.914 (−0.015)	0.845 (0.042)	0.015 (0.360)
Neuroimaging measures			

GM volume	0.765 (−0.064)	0.881 (−0.038)	0.430 (−0.014)
Glucose metabolism	0.428 (0.128)	0.372 (0.209)	0.024 (0.319)
Amyloid load	0.528 (0.107)	0.015 (0.496)	0.359 (−0.142)

**Anxiety symptoms**	**HC**	**SCD**	**ADC patients**

Cognitive measures			

Global cognition	0.484 (0.118)	*0.081* (*0.369)*	0.583 (0.079)
Episodic memory	0.655 (−0.070)	0.230 (−0.287)	0.221 (0.178)
Neuroimaging measures			

GM volume	0.961 (−0.011)	0.318 (−0.269)	0.818 (−0.038)
Glucose metabolism	0.272 (−0.192)	0.928 (−0.022)	0.619 (0.068)
Amyloid load	0.778 (−0.051)	0.440 (−0.184)	0.902 (−0.019)

*Values indicate p (r) values of the multiple linear regressions between depressive or anxiety symptoms on the one hand, and the corresponding cognitive or neuroimaging variables on the other hand for each group. All analyses with depressive and anxiety symptoms were corrected for level of education, age and sex. Values in bold correspond to significant p values (p < 0.05) and values in italic correspond to trends (0.05 < p < 0.1). ADC, Alzheimer’s continuum; GM, Gray matter; HC, Healthy controls; SCD, Subjective cognitive decline.*

As for anxiety symptoms, a trend for a positive association was found with global cognition within the SCD group (Table 2), as well as when also correcting for depressive symptoms (*p* = 0.06; see Supplementary Table 1). This association became significant with non-parametric analyses (*p* = 0.03; see Supplementary Table 2).

No association was found in the group of healthy controls with either depressive or anxiety symptoms (Table 2 and Supplementary Tables 1–3).

### Additional Analyses

#### Depressive Symptoms, Cognitive Performance and Awareness of Cognitive Deficits in ADC Patients

The positive relationship between episodic memory performance and depressive symptoms in ADC patients was not expected. Interestingly, a study showing similar results suggested that awareness of one’s cognitive deficits could be associated with worsened mood (Cerbone et al., 2020).

Along this line, we hypothesized that our finding with episodic memory might reflect anosognosia, such that ADC patients at a more advanced cognitive stage (i.e., with lower episodic memory performance) would be more anosognosic, either about their depressive symptoms (so that they have a lower depression score because they are less aware of their depressive symptoms); or about their cognitive deficits (so that they are less depressed about those). We thus ran additional analyses within the ADC patient group to test this hypothesis. We computed two composite delta scores of anosognosia for each ADC patient (Perrotin et al., 2015), one for global cognition and one for episodic memory performance, corresponding to the difference between the z-score of objective performance and the reversed z-score of subjective assessment [using the Cognitive Difficulties Scale (CDS) (McNair and Kahn, 1983; Kuhn et al., 2019)] for either global cognition (MMSE) or episodic memory (ESR) (see Supplementary Material 2 for further details). To test our hypothesis, we assessed the link between these two delta scores and depressive symptoms with multiple regressions corrected for level of education, age and sex, within the ADC patients. We found a positive association between the delta scores (awareness of cognitive and memory difficulties) and depressive symptoms (*p* = 0.01 and *p* < 0.001, respectively), as well as objective performance in global cognition (*p* < 0.001) and episodic memory (*p* < 0.001). This suggests that ADC patients with better cognitive and/or memory performance, are more aware of their cognitive difficulties and show more depressive symptoms; or reversely, that those with greater cognitive/memory deficits are more anosognosic and report fewer depressive symptoms.

### Voxelwise Brain Substrates of Depressive Symptoms in SCD and ADC Patients

We aimed to further investigate the brain substrates specifically involved in the positive associations found in the main analyses (Section “Psychoaffective factors and their links with cognition and neuroimaging biomarkers”) between depressive symptoms and both amyloid load in SCD patients and glucose metabolism in ADC patients. For this purpose, we performed voxelwise multiple regression analyses in SPM12 between depressive symptoms and amyloid deposition in SCD patients on the one hand, and glucose metabolism in ADC patients on the other hand. All analyses were corrected for education, age and sex. Results were evaluated for significance at p_uncorrected_ < 0.005 combined with a minimum cluster size determined by Monte-Carlo simulations using the AFNI’s 3dClustSim program to achieve a corrected statistical significance of *p* < 0.05.

In the SCD group, we found that higher depressive symptoms were associated with higher amyloid deposition mainly in the bilateral medial prefrontal, temporo-parietal, temporal, insular cortices and the right hippocampus (Figure 2B1).

In the ADC group, we found that higher depressive symptoms were associated with higher glucose metabolism in the bilateral precuneus, posterior cingulate—retrosplenial area, left temporal, superior parietal and temporal regions and the left hippocampus (Figure 2B2).

## Discussion

This study is, to our knowledge, the first to assess the links of anxiety and depressive symptoms with multiple AD-relevant indices, including cognitive and neuroimaging measures (i.e., global cognition, episodic memory, GM atrophy, glucose metabolism and amyloid deposition), in SCD and Alzheimer’s continuum. Altogether, we showed that SCD and ADC patients had higher depressive symptoms compared to healthy elders, which were associated with higher amyloid pathology in SCD, and with higher episodic memory performance and glucose metabolism in ADC patients.

In this study, we found no difference between groups regarding anxiety symptoms, but higher depressive symptoms in SCD and ADC patients compared to controls. This is in line with a previous study showing a higher frequency of depressive symptoms in clinical MCI and AD patients compared to controls, while anxiety symptoms were only different between mild and severe AD (Fernández-Martínez et al., 2010). Additionally, we found no association of anxiety symptoms with cognition and neuroimaging data, which is at odds with previous studies showing significant relationships with GM atrophy (Tagai et al., 2014; Hayata et al., 2015) and glucose hypometabolism (Hashimoto et al., 2006) in AD. This might be due to the fact that, in the present study, we focused on state measures of either anxiety or depressive symptoms, while these previous studies assessed trait anxiety. In addition, in these previous studies the level of anxiety symptoms of the patients was measured through an interview with their caregiver, while in our study it was self-rated by the patient. Thus, the differences in results could reflect discrepancies between self-rated and informant-rated anxiety.

### SCD Group

In the SCD group, we found that depressive symptoms were not related to objective cognitive/memory performance but were associated with higher amyloid load mainly in medial prefrontal and temporo-parietal regions, which are among the earliest regions affected by amyloid deposition in AD (Grothe et al., 2016, Grothe et al., 2017). While some previous studies found higher depressive symptoms (Balash et al., 2013; Buckley et al., 2013), and higher amyloid load (Amariglio et al., 2012, 2015; Perrotin et al., 2012, 2017; Snitz et al., 2015) associated with subjective cognitive decline, no study to date formally assessed the links between depressive symptoms and amyloid deposition, in this specific population. Our finding of an association between depressive symptoms with amyloid load in SCD could indicate that depressive symptoms represent a manifestation of the ongoing pathology and/or are a risk factor promoting amyloid accumulation, or that both depressive symptoms and amyloid plaques are caused by a common, yet unknown, factor.

Furthermore, we found that this association between depressive symptoms and amyloid was specific to this SCD stage, where there is subjective but no objective cognitive deficit, as it was neither found in the HC group nor in the MCI/AD patients, even when not selected for being amyloid-positive. Previous findings were conflicting as, in cognitively unimpaired elders (corresponding to our HC), some (Krell-Roesch et al., 2018) but not all (Donovan et al., 2015) studies found a relationship between depressive symptoms and amyloid load. Similarly, while some studies reported a link between depressive symptoms and amyloid load in MCI (Brendel et al., 2015; Krell-Roesch et al., 2019), a recent systematic review showed that most studies in fact did not find this association (Banning et al., 2019).

Recent criteria for SCD and updates about this concept have highlighted that SCD with biomarker evidence for AD have increased risk for future cognitive decline (Jessen et al., 2014, 2020). As we found depressive symptoms to be associated with amyloid deposition in SCD patients in our study, this suggests that SCD patients with depressive symptoms may be at even greater risk for cognitive decline. Altogether, this suggests that depressive symptoms in SCD is not a mere psychological consequence of their memory concern, but might be considered as an additional risk factor, and thus treated, in this population.

### Alzheimer’s Continuum

In the ADC patient group, we found depressive symptoms to be associated with better episodic memory performance and higher glucose metabolism, i.e., with a less advanced clinical and neurodegenerative stage of the disease. Previous studies showed conflicting findings; thus, one study found worse cognitive performance to be associated with depressive symptoms, but they assessed a mixed group of cognitively healthy elders and MCI patients (Zhang et al., 2020). Another study found no association between depressive symptoms and global cognition in either MCI or AD patients (Fernández-Martínez et al., 2010). However, our findings are in line with recent studies showing higher glucose metabolism in frontal regions and the fusiform gyrus in MCI patients with depressive symptoms (Auning et al., 2015; Brendel et al., 2015). In contrast to the preclinical stage (SCD), the emergence of anosognosia (i.e., progressive decrease in awareness of cognitive deficits) is known to occur as the disease progresses from MCI to AD stages (Hanseeuw et al., 2020; Vannini et al., 2020; Bastin et al., 2021; Cacciamani et al., 2021a,b). In these patients, the presence of anosognosia might impact their neuropsychiatric symptoms or ability to report those. Indeed, a recent study found that MCI patients with lower depression scores showed steeper decline in dementia severity measures compared to those with higher depression scores (Cerbone et al., 2020). The authors suggested that being aware of one’s cognitive deficits could be associated with worsened mood (more depressive symptoms), while, conversely, MCI patients who are less or not aware of their cognitive deficits may be less affected and show less to no depressive symptoms. Alternatively, it is also possible that patients at a more advanced stage of the disease (i.e., with more cognitive deficits) tend to report fewer depressive symptoms as they are less aware of their symptoms (more anosognosic). In line with these interpretations, depressive symptoms were found to be associated with less anosognosia in a group of patients with AD dementia (Kashiwa et al., 2005). In addition, the functional neural substrates of depressive symptoms in ADC patients were localized in regions which are known to be related to anosognosia in MCI and AD patients. Indeed, previous works highlighted that greater anosognosia in these patients was associated with reduced glucose metabolism in the posterior cingulate cortex, hippocampus, superior temporal and parietal gyri (Salmon et al., 2006; Nobili et al., 2010; Perrotin et al., 2015; Vannini et al., 2017; Hallam et al., 2020). The fact that we found higher depressive symptoms to be associated with better awareness of cognitive difficulties in our ADC patient group is also in line with those hypotheses.

In contrast to cognition and glucose metabolism, we found no link between depressive symptoms and GM volume nor amyloid deposition in ADC patients. Previous findings are conflicting, with studies also reporting no links (Bruen et al., 2008; Starkstein et al., 2009; Berlow et al., 2010; Mori et al., 2014; Huey et al., 2017) while others found a positive association (Auning et al., 2015; Enache et al., 2015), or a negative (Lebedeva et al., 2014; Wu et al., 2020) with depressive symptoms in MCI or AD populations. Our findings suggest that glucose metabolism appears to be more strongly associated with depressive symptoms in amyloid-positive MCI-AD patients than GM volume or amyloid deposition.

### Methodological Considerations and Perspectives

Even though the MADRS has been shown to be a good measure of depression relatively independent from dementia severity (Müller-Thomsen et al., 2005), as our participants were selected for the lack of clinically significant anxiety or depression, one of the limitations of our study refers to the skewed distribution of MADRS, with 42% of the total participants reporting depressive symptoms. To limit the bias associated with the low variability and thus low power of analyses, we repeated all analyses with non-parametric tests, as well as in subgroups including only individuals with at least one depressive symptom.

Our study, assessing multiple hallmarks of AD (i.e., global cognition, episodic memory, gray matter volume, glucose metabolism and amyloid load), was cross-sectional in design and thus couldn’t assess the causality and direction of these links. Future longitudinal analyses would allow to investigate this question by examining the links between baseline levels and changes over time in depressive and anxiety symptoms and neuroimaging biomarkers. Similarly, as discussed above, we investigated state measures of depressive and anxiety symptoms as they represent states at a given time that we expect to easily change over time and be modifiable through treatment/interventions. Moreover, anxiety symptoms were assessed using a self-report questionnaire; as it is subjective, the measure could be biased by the subject’s honesty, awareness and introspective ability.

## Conclusion

This study showed that depressive symptoms were associated with higher amyloid load in SCD, and with better episodic memory and higher glucose metabolism in ADC patients. Overall, our findings suggest that depressive symptoms reflect distinct processes along the course of AD, with higher symptoms reflecting greater likelihood of AD biomarker at the SCD stage, while, conversely, they would reflect greater awareness of cognitive deficits associated with less severe cognitive stage of the disease in ADC patients. Thus, this study shows the relevance of assessing and following depressive symptoms in SCD, and to manage them in cognitively impaired patients, to improve the prevention as well as the prognosis and quality of life of both patients and caregivers.

## Data Availability Statement

The datasets presented in this article are not readily available because IMAP data are made available upon request to the sponsor (Caen University Hospital) and the principal investigator. Requests to access the datasets should be directed to GC, chetelat@cyceron.fr.

## Ethics Statement

The studies involving human participants were reviewed and approved by Comité de Protection des Personnes Nord-Ouest III. The patients/participants provided their written informed consent to participate in this study.

## Author Contributions

IM and GC contributed to the conception and design of the study. IM, ET, and GC contributed to the acquisition, analysis, or interpretation of the data and wrote the draft manuscript. IM, ET, FM, SD, VD, DV, NM, GP, and GC contributed to the critical revision of the manuscript for important intellectual content. IM performed the statistical analysis. GC obtained the funding. FM, DV, and GP contributed to the administrative, technical, or material support. GC and VD were the principal investigators of the IMAP + research protocol. All authors took public responsibility for the whole or part of the content, contributed to the acquisition, analysis and interpretation of data, manuscript revision, and read and approved the submitted version.

## Conflict of Interest

GP was a member of the DSMB of the Age-well trial for the Inserm Partner (not paid). GC was Member of the External Advisory Board (EAB) of the Lifebrain H2020 European project and of the Operational Committee of the Foundation Plan Alzheimer (personal fees) and of the Imaging Scientific Advisory Groups of European Prediction of Alzheimer’s Disease (EPAD) Consortium, EU (not paid). The remaining authors declare that the research was conducted in the absence of any commercial or financial relationships that could be construed as a potential conflict of interest.

## Publisher’s Note

All claims expressed in this article are solely those of the authors and do not necessarily represent those of their affiliated organizations, or those of the publisher, the editors and the reviewers. Any product that may be evaluated in this article, or claim that may be made by its manufacturer, is not guaranteed or endorsed by the publisher.
