# Supplementary material for: Depressive Symptoms Have Distinct Relationships With Neuroimaging Biomarkers Across the Alzheimer’s Clinical Continuum
Source: Front Aging Neurosci. 2022 Jun 20;14:899158. doi: 10.3389/fnagi.2022.899158 (PMC9251580; doi:10.3389/fnagi.2022.899158)
Supplement: Supplementary file 1 [file Table_1.DOCX]

Supplementary Data

# Supplementary Material

## Neuroimaging acquisition

High-resolution T1-weighted MRI anatomical images were acquired on a Philips Achieva 3.0 T scanner using a 3D fast-field echo sequence (3D-T1-FFE sagittal; repetition time = 20 ms; echo time = 4.6 ms; flip angle = 10°; 180 slices with no gap; slice thickness = 1 mm; field of view = 256 x 256 mm^2^; in-plane resolution = 1 x 1mm^2^).

Both FDG and florbetapir PET images were acquired with a Discovery RX VCT 64 PET-CT device (General Electric Healthcare) with a resolution of 3.76 x 3.76 x 4.9 mm^3^ (field of view = 157 mm). Forty-seven planes with a voxel size of 1.95 x 1.95 x 3.27 mm^3^ were obtained and an attenuation correction was performed before the PET acquisition with a transmission scan.

FDG-PET acquisitions were performed after at least 6 hours of fasting, and after 30 minutes of rest in a quiet and dark room. Fifty minutes after intravenous injection of ~180 MBq of FDG, the participants underwent a 10-minute scan. Florbetapir-PET scan was acquired for each participant 50 minutes after the intravenous injection of ~4 MBq/kg of florbetapir, for a duration of 20 minutes, except for two SCD, and three ADC patients, who had a 10-minute acquisition.

## Additional analyses – Delta scores reflecting cognitive awareness

The subjective measure of global cognitive difficulties was the Cognitive Difficulties Scale (CDS) (McNair and Kahn, 1983; Kuhn et al., 2019), and a subscore of 12 items from the CDS was used as an assessment of subjective memory difficulties, as previously described elsewhere (La Joie et al., 2016). Raw scores were converted to z-scores using the mean and standard deviation from the HC group as a reference. The subjective scores were reversed so that a high score reflected a high estimated level of performance, and the two z-scores were then subtracted from one another (z-score objective – reversed z-score subjective). This way, a higher (more positive) delta score indicated greater awareness of cognitive/memory deficits, while a lower (more negative) delta score indicated more severe anosognosia (Perrotin et al., 2015; Vannini et al., 2017).

# Supplementary Tables

## Supplementary Table 1

| Depressive symptoms | HC | SCD | ADC patients |
| --- | --- | --- | --- |
| Cognitive measures |  |  |  |
| Global cognition | 0.313 (-0.163) | 0.255 (0.260) | 0.112 (0.240) |
| Episodic memory | 0.505 (0.103) | 0.725 (-0.038) | **0.012 (0.373)** |
| Neuroimaging measures |  |  |  |
| GM volume | 0.462 (-0.160) | 0.930 (-0.027) | 0.440 (-0.135) |
| Glucose metabolism | 0.481 (0.121) | 0.244 (0.309) | **0.032 (0.301)** |
| Amyloid load | 0.422 (0.139) | **0.008 (0.657)** | 0.279 (-0.171) |
| Anxiety symptoms | HC | SCD | ADC patients |
| Cognitive measures |  |  |  |
| Global cognition | 0.371 (0.150) | *0.060 (0.390)* | 0.914 (0.016) |
| Episodic memory | 0.506 (-0.106) | 0.226 (-0.294) | 0.577 (0.088) |
| Neuroimaging measures |  |  |  |
| GM volume | 0.954 (0.013) | 0.329 (-0.267) | 0.985 (-0.003) |
| Glucose metabolism | 0.212 (-0.220) | 0.949 (0.016) | 0.930 (-0.012) |
| Amyloid load | 0.648 (-0.082) | 0.594 (-0.160) | 0.868 (0.025) |

**Supplementary Table 1: Relationships of psychoaffective factors to cognition and neuroimaging measures, corrected for education, age, sex and anxiety or depressive symptoms.** Values indicate p (r) values of the multiple linear regressions between depressive or anxiety symptoms on the one hand, and the corresponding cognitive or neuroimaging variables on the other hand for each group. All analyses with depressive symptoms were corrected for level of education, age, sex and anxiety symptoms while all analyses with anxiety symptoms were corrected for level of education, age, sex and depressive symptoms. Values in bold correspond to significant p values (p<0.05) and values in italic correspond to trends (0.05<p<0.1). Abbreviations *ADC,* Alzheimer’s continuum; *GM,* Gray matter; *HC,* Healthy controls; *SCD,* Subjective cognitive decline.

## Supplementary Table 2

| Depressive symptoms | HC | SCD | ADC patients |
| --- | --- | --- | --- |
| Cognitive measures |  |  |  |
| Global cognition | 0.401 (-0.117) | 0.185 (0.236) | 0.368 (0.124) |
| Episodic memory | 0.996 (0.001) | 0.930 (-0.015) | *0.074 (0.258)* |
| Neuroimaging measures |  |  |  |
| GM volume | **0.049 (-0.275)** | 0.480 (-0.139) | 0.471 (-0.099) |
| Glucose metabolism | 0.116 (0.230) | 0.698 (0.078) | *0.073 (0.246)* |
| Amyloid load | 0.279 (0.169) | *0.071 (0.353)* | 0.456 (-0.102) |
| Anxiety symptoms | HC | SCD | ADC patients |
| Cognitive measures |  |  |  |
| Global cognition | 0.776 (0.043) | **0.030 (0.411)** | 0.572 (-0.079) |
| Episodic memory | 0.961 (0.007) | 0.873 (-0.031) | 0.162 (0.205) |
| Neuroimaging measures |  |  |  |
| GM volume | 0.433 (-0.116) | 0.243 (-0.242) | 0.419 (0.113) |
| Glucose metabolism | 0.928 (-0.014) | 0.404 (0.179) | 0.987 (0.002) |
| Amyloid load | 0.982 (-0.004) | 0.404 (0.179) | 0.489 (0.097) |

**Supplementary Table 2: Non-parametric associations of psychoaffective factors with cognition and neuroimaging measures.** Values indicate p (r) values of Spearman’s correlations between depressive or anxiety symptoms on the one hand, and the corresponding cognitive or neuroimaging variables on the other hand for each group. Values in bold correspond to significant p values (p<0.05) and values in italic correspond to trends (0.05<p<0.1). Abbreviations *ADC,* Alzheimer’s continuum; *GM,* Gray matter; *HC,* Healthy controls; *SCD,* Subjective cognitive decline.

## Supplementary Table 3

| Non-parametric | HC | SCD | ADC patients |
| --- | --- | --- | --- |
| Cognitive measures |  |  |  |
| Global cognition | 0.206 (-0.295) | 0.227 (0.268) | 0.259 (0.181) |
| Episodic memory | 0.784 (-0.065) | 0.131 (0.325) | 0.493 (0.118) |
| Neuroimaging measures |  |  |  |
| GM volume | 0.652 (0.111) | 0.183 (0.339) | 0.752 (0.051) |
| Glucose metabolism | 0.932 (-0.021) | 0.699 (0.105) | 0.244 (0.188) |
| Amyloid load | 0.971 (0.009) | **0.016 (0.592)** | 0.468 (-0.117) |
| corrected for the level of education, age and sex | HC | SCD | ADC patients |
| Cognitive measures |  |  |  |
| Global cognition | 0.377 (-0.192) | 0.287 (0.278) | 0.102 (0.284) |
| Episodic memory | 0.801 (-0.054) | 0.221 (0.345) | 0.157 (0.253) |
| Neuroimaging measures |  |  |  |
| GM volume | 0.136 (0.442) | 0.703 (0.138) | 0.453 (-0.147) |
| Glucose metabolism | *0.071 (-0.516)* | 0.147 (0.505) | **0.039 (0.331)** |
| Amyloid load | 0.737 (-0.085) | **0.036 (0.584)** | 0.166 (-0.252) |
| corrected for the level of education, age, sex and anxiety symptoms | HC | SCD | ADC patients |
| Cognitive measures |  |  |  |
| Global cognition | 0.280 (-0.256) | 0.107 (0.503) | 0.117 (0.286) |
| Episodic memory | 0.955 (-0.014) | 0.494 (0.231) | 0.998 (0.306) |
| Neuroimaging measures |  |  |  |
| GM volume | 0.261 (0.358) | 0.545 (0.273) | 0.586 (-0.113) |
| Glucose metabolism | 0.211 (-0.412) | 0.101 (0.750) | **0.040 (0.340)** |
| Amyloid load | 0.795 (0.073) | *0.059 (0.748)* | 0.174 (-0.261) |

**Supplementary Table 3: Relationships of depressive symptoms with cognition and neuroimaging measures in subgroups with at least one depressive symptom (n=21 HC, n=23 SCD, n=42 ADC patients).** Values indicate p (r) values of the multiple linear regressions between depressive and the corresponding cognitive or neuroimaging variables for each group. Values in bold correspond to significant p values (p<0.05) and values in italic correspond to trends (0.05<p<0.1). Abbreviations *ADC,* Alzheimer’s continuum; *GM,* Gray matter; *HC,* Healthy controls; *SCD,* Subjective cognitive decline.

## Supplementary Table 4

|  | MCI and AD patients  (Aβ positive and negative) | n |
| --- | --- | --- |
| Sex, n (M/F) | 74 (43/31) |  |
| Level of education, years | 11.00 (3.58) | 74 |
| Age, years | 71.49 (8.50) | 74 |
| Aβ positive, n (%) | 56 (75.68) | 74 |
| Amyloid load | 1.34 (0.37) | 74 |
| Psychoaffective measures |  |  |
| Depressive symptoms | 3.33 (3.67) | 73 |
| Anxiety symptoms | 29.99 (10.58) | 71 |
| Cognitive measures |  |  |
| Global cognition | 24.28 (4.42) | 74 |
| Episodic memory | 6.38 (2.84) | 68 |

**Supplementary Table 4: Characteristics of the group of MCI (n=42) and AD (n=32) amyloid-positive and amyloid-negative patients.** Values indicate mean (standardized deviation) unless otherwise stated. Abbreviations *Aβ,* Amyloid.

# References

Kuhn, E., Moulinet, I., Perrotin, A., La Joie, R., Landeau, B., Tomadesso, C., et al. (2019). Cross-sectional and longitudinal characterization of SCD patients recruited from the community versus from a memory clinic: subjective cognitive decline, psychoaffective factors, cognitive performances, and atrophy progression over time. *Alzheimers Res Ther* 11, 61. doi:10.1186/s13195-019-0514-z.

La Joie, R., Perrotin, A., Egret, S., Pasquier, F., Tomadesso, C., Mézenge, F., et al. (2016). Qualitative and quantitative assessment of self-reported cognitive difficulties in nondemented elders: Association with medical help seeking, cognitive deficits, and β-amyloid imaging. *Alzheimers Dement (Amst)* 5, 23–34. doi:10.1016/j.dadm.2016.12.005.

McNair, D., and Kahn, R. (1983). Self-assessment of cognitive deficits. Assessment in Geriatric Psychopharmacology. *Assessment in Geriatric Psychopharmacology*, 119–36.

Perrotin, A., Desgranges, B., Landeau, B., Mézenge, F., Joie, R. L., Egret, S., et al. (2015). Anosognosia in Alzheimer disease: Disconnection between memory and self-related brain networks. *Annals of Neurology* 78, 477–486. doi:10.1002/ana.24462.

Vannini, P., Hanseeuw, B., Munro, C. E., Amariglio, R. E., Marshall, G. A., Rentz, D. M., et al. (2017). Anosognosia for memory deficits in mild cognitive impairment: Insight into the neural mechanism using functional and molecular imaging. *Neuroimage Clin* 15, 408–414. doi:10.1016/j.nicl.2017.05.020.
